# Green Preparation and Antibacterial Activity Evaluation of AgNPs-*Blumea balsamifera* Oil Nanoemulsion

**DOI:** 10.3390/molecules29092009

**Published:** 2024-04-26

**Authors:** Chunfang Ma, Bingnan Liu, Lingfeng Du, Wei Liu, Yue Zhu, Teng Chen, Zuhua Wang, Hongpeng Chen, Yuxin Pang

**Affiliations:** 1 College of Chinese Medicine Resources, Guangdong Pharmaceutical University, Yunfu 527325, China; m16653194486@163.com (C.M.); lbn0714@163.com (B.L.); dlingf00@163.com (L.D.); l_w_biophilia@163.com (W.L.); 2 College of Pharmaceutical Sciences, Guizhou University of Traditional Chinese Meidicine, Guiyang 550025, China; chenteng0907@163.com (T.C.); wangrui551601@163.com (Z.W.); 3 Nano-Drug Technology Research Center of Guizhou University of Traditional Chinese Medicine, Guiyang 550025, China; 4 Yunfu Traditional Chinese Medicine Resources and Germplasm Resources Database Management Center, Yunfu 527325, China

**Keywords:** *Blumea balsamifera* oil, tea saponin, nanoemulsion, silver nanoparticles, synergistic antibacterial activity, ultrasonic emulsification

## Abstract

Bacterial infection is a thorny problem, and it is of great significance to developing green and efficient biological antibacterial agents that can replace antibiotics. This study aimed to rapidly prepare a new type of green antibacterial nanoemulsion containing silver nanoparticles in one step by using *Blumea balsamifera* oil (BBO) as an oil phase and tea saponin (TS) as a natural emulsifier and reducing agent. The optimum preparation conditions of the AgNPs@BBO-TS NE were determined, as well as its physicochemical properties and antibacterial activity in vitro being investigated. The results showed that the average particle size of the AgNPs@BBO-TS NE was 249.47 ± 6.23 nm, the PDI was 0.239 ± 0.003, and the zeta potential was −35.82 ± 4.26 mV. The produced AgNPs@BBO-TS NE showed good stability after centrifugation and 30-day storage. Moreover, the AgNPs@BBO-TS NE had an excellent antimicrobial effect on *Staphylococcus aureus*, *Escherichia coli*, and *Pseudomonas aeruginosa*. These results demonstrated that the AgNPs@BBO-TS NE produced in this study can be used as an efficient and green antibacterial agent in the biomedical field.

## 1. Introduction

Bacterial infection has posed a serious threat to human health, as if it is not prevented, controlled, and treated promptly, it can progress into a severe infectious disease [1]. Taking antibiotics is one of the most common methods for treating various infectious diseases. However, the long-term irrational use of antibiotics can increase bacterial resistance and even lead to the emergence of superbugs that are resistant to multiple antibiotics at the same time [2,3]. Therefore, developing an efficient and green antimicrobial agent to resist the invasion of microorganisms is of great significance for the protection of human health [4].

Plant essential oils (EOs) are lipophilic and volatile liquid extracts that are obtained from plants, which are considered to be a kind of potential antibacterial agent [5]. *Blumea balsamifera* oil (BBO) is extracted and isolated from the leaves of *Blumea balsamifera* (L.) *DC*, and its main components are β-pinene, D-camphor, L-camphor, β-carydene, and so on [6,7]. Yang et al. [8] found that BBO had outstanding antibacterial activity against *S. aureus* by its destroying the cell wall structure of bacteria. Sakee et al. [9] showed that BBO affected the whole cell level, protein level, and mRNA level of *Haemophilus parahaemus*, and showed a significant antibacterial effect even at low doses. However, the application of BBO is often limited by its high volatility, low water solubility, and chemical instability [6]. Hence, a suitable method is needed to enhance the antibacterial activity of EOs. To date, studies had found that the EOs encapsulated in nanoemulsions exhibit great superiority in their better physical stability and improved antibacterial activity over equivalent EOs [10]. A nanoemulsion is a nanostructured system with a particle size between 20 and 500 nm, which is composed of a water phase, an oil phase, and a surfactant [11]. By carefully selecting the composition of the oil phase, water phase, and surfactant, a new type of green antibacterial nanoemulsion with good antibacterial activity can be constructed. For example, when designing a prescription for a nanoemulsion, an oil phase with antibacterial activity (such as BBO) [8] and/or an emulsifier (such as TS) [12], or added lipophilic antimicrobials (such as polyphenols) were often chosen [13].

In addition, nanoemulsions may carry different antimicrobials at different locations to achieve synergistic effects [13]. Silver has a longer history in the antibacterial field, and is widely used in medicine, food, and other fields because of its good bactericidal effect, low toxicity, and difficulty in accumulating in the body [14]. AgNPs with an average particle size of <100 nm have attracted more continuous attention due to their having more excellent antibacterial properties than free silver ions [15]. To overcome the limitations of physical and chemical procedures, more and more hopeful green synthesis methods have been developed [16]. In recent years, researchers have found that the plant extracts-mediated preparation of NPs was one of the green ways to synthesize AgNPs [17]. Yang et al. [18] reported that the antibacterial activity of AgNPs and *Lonicera japonica Thunb* extracts were significantly higher than that of pure AgNPs or herbal extracts. Phan et al. [16] also found that the two herbal extracts of *Allium sativum* and *Phyllanthus urinaria* can be used to synthesize AgNPs, and the combination of herb antibiotics and AgNPs showed an antibacterial effect on *E. coli* in vitro. TS, a glycoside compound extracted from *Camellia* seeds, is not only a natural non-ionic surfactant with excellent properties that can improve the solubility and bioavailability of insoluble drugs, but also has various pharmacological activities such as the inhibition of bacterial growth, anti-oxidation, and anti-inflammation [19,20]. Therefore, TS may have the potential to be used as a natural surfactant and a reducing agent to synthesize nanoemulsions and AgNPs, respectively.

**Figure 1:**
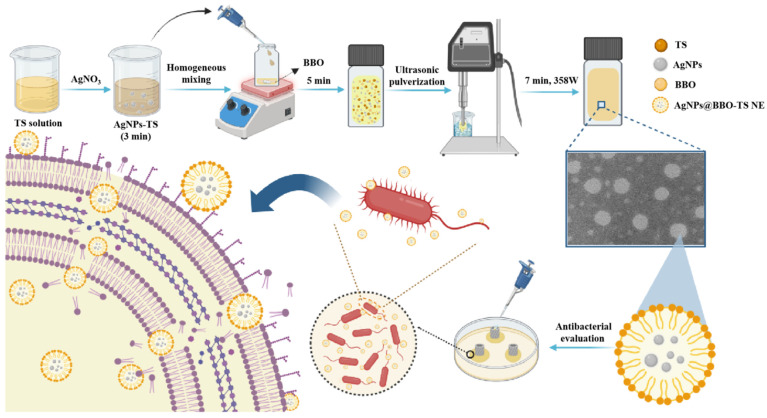
The preparation and an antibacterial schematic diagram of the AgNPs@BBO-TS NE.

In this study, BBO was used as an oil phase and TS was used as a natural emulsifier to prepare a green antibacterial nanoemulsion (BBO-TS NE) using a high-energy emulsification method, and its preparation conditions were optimized to determine the best prescription and process parameters of nanoemulsion. Considering that TS had good antioxidant activity, we also tried to use TS as a reducing agent to prepare a AgNPs@BBO-TS NE in one step, and its physicochemical properties, stability, cytotoxicity, and antibacterial activity in vitro were systematically investigated (Figure 1).

## 2. Results

### 2.1. Single-Factor Experiments to Optimize the Prescription of a BBO-TS NE

#### 2.1.1. Effect of BBO Content

**Figure 2:**
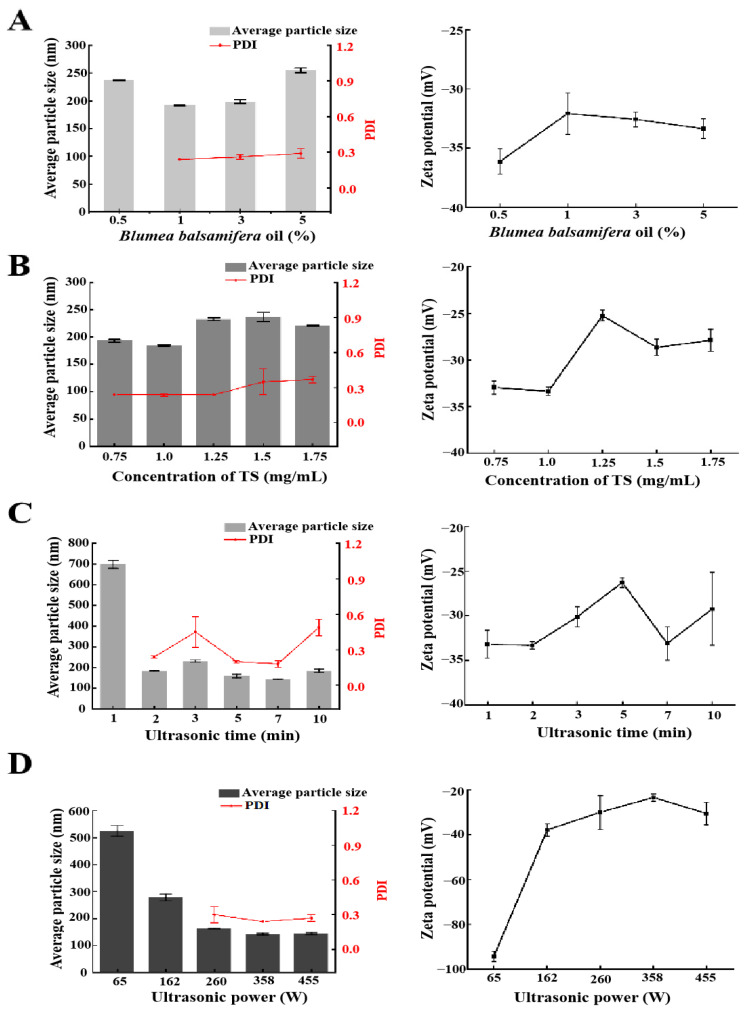
The effects of different variables on the average particle size, PDI, and zeta potential of the BBO-TS NE: different BBO contents (**A**), TS concentrations (**B**), ultrasonic times, (**C**) and ultrasonic powers (**D**).

BBO is a pure plant green antibiotic, and many studies have shown that it has significant pharmacological activities [7,21]. In this study, BBO was selected as a natural oil phase to prepare a nanoemulsion, and different contents of BBO (volume fractions of 0.5%, 1%, 3%, and 5%) were studied. As shown in Figure 2A, the particle size, PDI, and zeta potential of the BBO-TS NE significantly changed when the BBO content increased from 0.5% to 5%. When the content of the BBO varied from 0.5% to 1%, the average particle size of the BBO-TS NE decreased to be the lowest (191.6 ± 0.8 nm), and the PDI also appeared. When the amount of the BBO varied from 1% to 5%, the average particle size and the PDI of the BBO-TS NE increased in a dose-dependent manner, the average particle size increased from 191.6 ± 0.8 nm to 254.8 ± 4.3 nm, and the PDI increased from 0.24 to 0.29. In addition, the absolute values of the zeta potentials were all greater than 30 mV, which met the requirement of the potential value for nanoemulsion stabilization. According to these results from the average particle size and DLS, the BBO with 1% was selected for subsequent experiments.

#### 2.1.2. Effect of TS Concentration

Surfactants are very important to promote the formation of a nanoemulsion. It is reported that the proper concentration of surfactant has a positive regulatory effect on the characteristics of a nanoemulsion [22]. TS is a natural surfactant with a hydrophilic and hydrophobic structure, which has the functions of emulsification, wetting, and dispersion [23,24]. The particle size of the BBO-TS NE was decreased when the concentration of TS increased from 0.75 mg/mL to 1.0 mg/mL, and then increased to above 200 nm when the concentration of TS was higher than 1.25 mg/mL. The PDI value of the BBO-TS NE was stable at 0.24 when the concentration of TS increased from 0.75 mg/mL to 1.25 mg/mL, but it increased constantly when the concentration of TS reached 1.5 mg/mL and higher than 0.3 (Figure 2B). The optimal concentration of TS was determined to be at 1 mg/mL, because the particle size of the BBO-TS NE was the smallest (184.3 nm), the PDI was the lowest (0.24), and the absolute values of the zeta potentials were the highest (|−33.33 ± 0.43 mV|) at this concentration.

#### 2.1.3. Influence of Ultrasonic Power and Ultrasonic Time

The ultrasonic process can provide the required energy in the shortest time and obtain a uniform fluid with the smallest droplet size. Ultrasonic power and ultrasonic time are primary factors affecting nanoemulsion performance [25]. Under the conditions of 1% BBO as an oil phase, a TS concentration of 1 mg/mL, and an ultrasonic power of 162 W, the particle size of the BBO-TS NE was the largest and the PDI value did not appear when the ultrasound duration was 1 min, while the particle size and PDI of the nanoemulsion showed a downward trend when the ultrasound duration was 3 to 7 min. It was worth noting that when the ultrasonic duration was 7 min, the zeta potential was −33.13 mV, and the minimum particle size and PDI of the nanoemulsion were reached, which were 143.9 ± 0.81 nm and 0.18 ± 0.03, respectively (Figure 2C). Therefore, the ultrasonic duration for the BBO-TS NE was determined to be 7 min. Finally, with other variables unchanged, we evaluated the effect of different amounts of ultrasonic power (65, 162, 260, 358, 455 W) on the BBO-TS NE. As shown in Figure 2D, with the increase of ultrasonic power from 65 to 358 W, the average particle size of the nanoemulsion decreased, and the particle size reached a minimum of 141.4 ± 2.75 nm at 358 W; among three different ultrasonic powers (260, 358, and 455 W), the corresponding PDI values of 260, 358, and 455 W were 0.30 ± 0.07, 0.24 ± 0.00, and 0.27 ± 0.03, separately. According to the results of the average particle size and PDI, the optimal ultrasonic power was set as 358 W.

### 2.2. The Existing Form of Silver in BBO-TS NE

**Figure 3:**
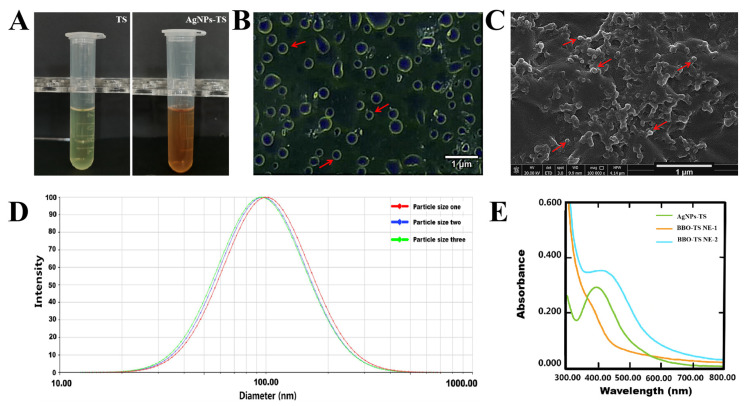
The color change before and after adding AgNO_3_ into the TS aqueous solution (**A**); an optical microscope image (**B**), a SEM image, (**C**) and the size distribution (**D**) of the AgNPs-TS; the UV–visible spectroscopy of AgNPs-TS, BBO-TS NE-1 (without silver), and BBO-TS NE-2 (add silver) (**E**). The red arrows of B and C point to AgNPs.

After adding AgNO_3_ into the TS aqueous solution, the color of the solution was changed from yellow to brown (Figure 3A), and we hypothesized that AgNPs were formed in this solution. To evaluate the presence of AgNPs, an optical microscope and SEM were first used for analysis. As shown in Figure 3B,C, smooth and spherical AgNP particles were found under the optical microscope and SEM. The elemental composition of the AgNPs was analyzed by EDS, as shown in Figure S1 and Table S1, and the content of the Ag element was 0.93%. The results of the DLS analysis showed that the particle’s average size was 97.07 ± 2.88 nm and the PDI was 0.255 ± 0.004, with a narrow distribution (Figure 3D). The UV–visible spectroscopy spectra in the region of 300 to 800 nm displayed a peak between 390 and 396 nm, which was attributed to the distinctive surface plasmon resonance peak of AgNPs, indicating that the formation of AgNPs in TS aqueous solution means it is a AgNPs-TS solution. To further verify that the silver in the BBO-TS NE still exists in the form of AgNPs, BBO-TS NEs with and without silver were analyzed using UV-visible spectroscopy. As shown in Figure 3E, BBO-TS NE-1 had no absorption peak in the wavelength range of 300 to 800 nm, while, after adding silver, the UV–visible spectrum of BBO-TS NE-2 showed a shift peak between 399 and 411 nm (at about 400 nm), which is the same as the peak position of the AgNPs-TS, proving that silver was loaded in the form of AgNPs in the BBO-TS NE (this was named the AgNPs@BBO-TS NE). As shown in Figure S2 and Table S2, through the EDS analysis of BBO-TS NE-2, it was found that there was a characteristic peak of the Ag element in BBO-TS NE-2, which indicated that silver was loaded in the form of AgNPs in the BBO-TS NE.

### 2.3. AgNO_3_ Concentration

**Figure 4:**
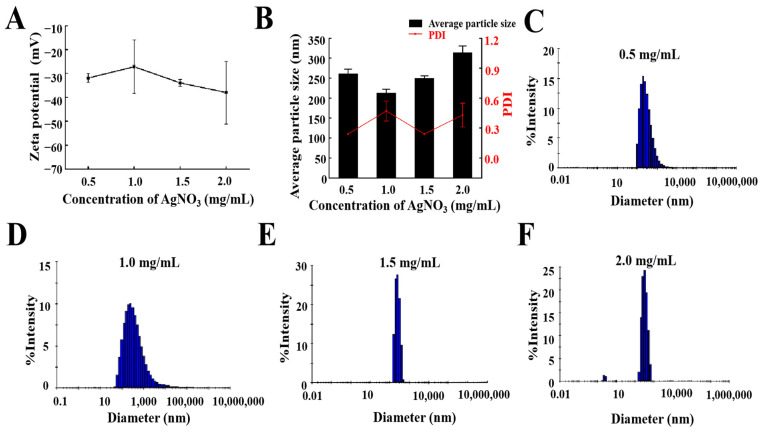
The zeta potential (**A**), average particle size and PDI (**B**), and a size distribution diagram (**C**–**F**) of the AgNPs@BBO-TS NE with different concentrations of AgNO_3_.

According to the optimal prescription and preparation process determined above, different concentrations of AgNO_3_ (0.5, 1, 1.5, and 2 mg/mL) were added, and the average particle size, PDI value, zeta potential, and particle size distribution were considered comprehensively. As shown in Figure 4B, when the concentration of AgNO_3_ was lower than 1.5 mg/mL, the particle size or PDI of the AgNPs@BBO-TS NE was larger, and when the concentration of AgNO_3_ was 2 mg/mL, the particle size was 313.9 ± 16.6 nm, and the PDI was 0.43; but, when the AgNO_3_ was 1.5 mg/mL, the average particle size of the AgNPs@BBO-TS NE was 249.47 ± 6.23 nm, and the PDI was 0.239. The absolute value of the zeta potential of the AgNPs@BBO-TS NE was first decreased and then increased as the concentration of AgNO_3_ varied from 0.5 mg/mL to 2 mg/mL; except for the concentration of 1 mg/mL, all of them were higher than 30 mV (Figure 4A). In addition, when the concentration of AgNO_3_ was 1.5 mg/mL, the droplet size distribution of the AgNPs@BBO-TS NE was the narrowest (Figure 4C–F). These results showed that 1.5 mg/mL of AgNO_3_ was the optimal concentration.

### 2.4. Characterization of the AgNPs@BBO-TS NE

#### 2.4.1. Appearance and Type Analysis

**Figure 5:**
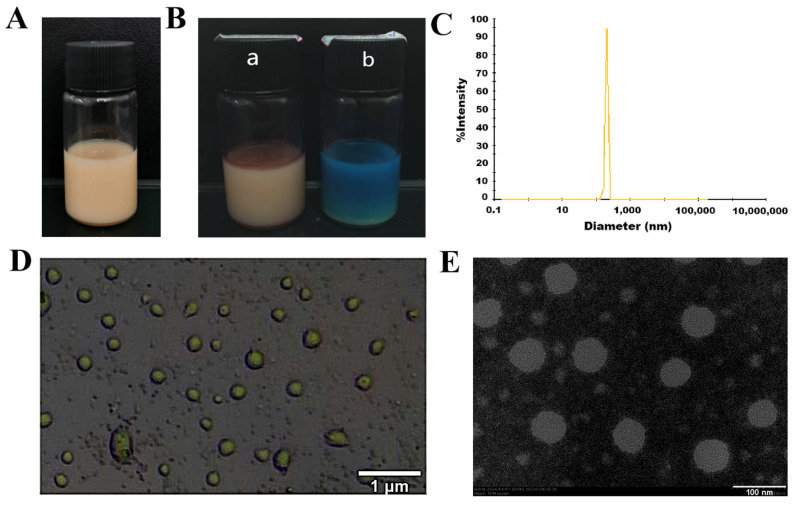
The appearance (**A**), Sudan III stain and methylene blue staining ((**B**), a is Sudan III; b is methylene blue), particle size distribution (**C**), an optical electron microscope image (**D**), and a transmission electron microscope image (**E**) of the AgNPs@BBO-TS NE.

As shown in Figure 5A, the AgNPs@BBO-TS NE had an excellent appearance, was a uniformly brown, milky liquid, without delamination, precipitation, and oil droplets floating. Water-soluble methylene blue and fat-soluble Sudan red dyes were used to identify the type of AgNPs@BBO-TS NE; if the Sudan red diffuses quickly, it is a W/O nanoemulsion; otherwise, it is O/W nanoemulsion [26]. As the diffusion speed of methylene blue was faster and more uniform (Figure 5B) in the AgNPs@BBO-TS NE, it could be classified as an O/W nanoemulsion.

#### 2.4.2. Average Particle Size, PDI, Zeta Potential, and Size Distribution

The average particle size, PDI, zeta potential, and particle size distribution are remarkable indicators of a nanoemulsion, which can describe the size, stability, uniformity, and dispersion of a nanoemulsion. The experimental results analyzed by the laser particle size/zeta potential analyzer are displayed in Table 1; the average particle size of the AgNPs@BBO-TS NE was 249.47 ± 6.23 nm, the PDI was 0.239 ± 0.003, and the zeta potential was −35.82 ± 4.26 mV. The particle size distribution of the AgNPs@BBO-TS NE is shown in Figure 5C; the peak width was very narrow, which indicated that the droplet size distribution was very uniform.

#### 2.4.3. Morphology, pH, and Turbidity

As shown in the optical microscope and TEM images, the droplets of the AgNPs@BBO-TS NE were entirely spherical, uniform in size, and well dispersed, and no phenomena such as aggregation and rupture were found (Figure 5D,E). The pH and turbidity of the AgNPs@BBO-TS NE were 4.09 ± 0.03 and 20.72 ± 0.09 cm^−1^, respectively.

### 2.5. Stability Studies

**Figure 6:**
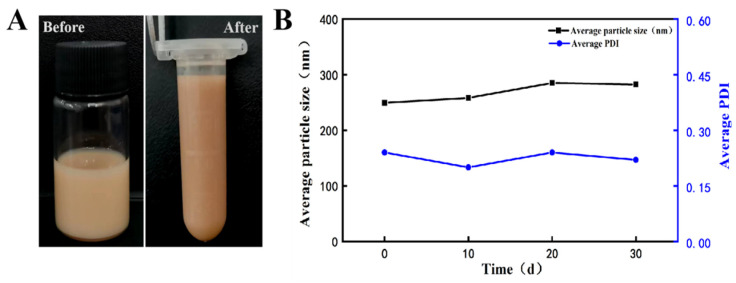
The appearance of the AgNPs@BBO-TS NE before and after centrifugation (**A**) and the effect of storage time on the average particle size and PDI of the AgNPs@BBO-TS NE (**B**).

The stability of the AgNPs@BBO-TS NE in terms of its phase separation, changes in average particle size, and PDI were evaluated via centrifugation and storage at room temperature for different durations. After being centrifugated for 15 min at 4500 rpm, the appearance of the AgNPs@BBO-TS NE did not change and no phase separation, creaming, or sedimentation was observed (Figure 6A). According to Table 2, the centrifugal stability constant *K* values were all greater than 90%, indicating that the AgNPs@BBO-TS NE had good centrifugal stability.

In addition, the long-term storage stability was studied by keeping the AgNPs@BBO-TS NE sample for 30 days at room temperature, and the average droplet size and PDI were analyzed every 10 days. As shown in Figure 6B, with the extension of storage time, the particle size of the AgNPs@BBO-TS NE generally increased slightly, but the increase was less than 35 nm, and the increasing trend in the PDI was also not evident, which stayed around 0.2. In detail, during the period of 10 to 20 d, the particle size and PDI of the AgNPs@BBO-TS NE increased slowly, then decreased on day 30 and closed at the initial values of 0 d. These results proved that the produced AgNPs@BBO-TS NE in this article had good storage stability.

### 2.6. Cytotoxicity

**Figure 7:**
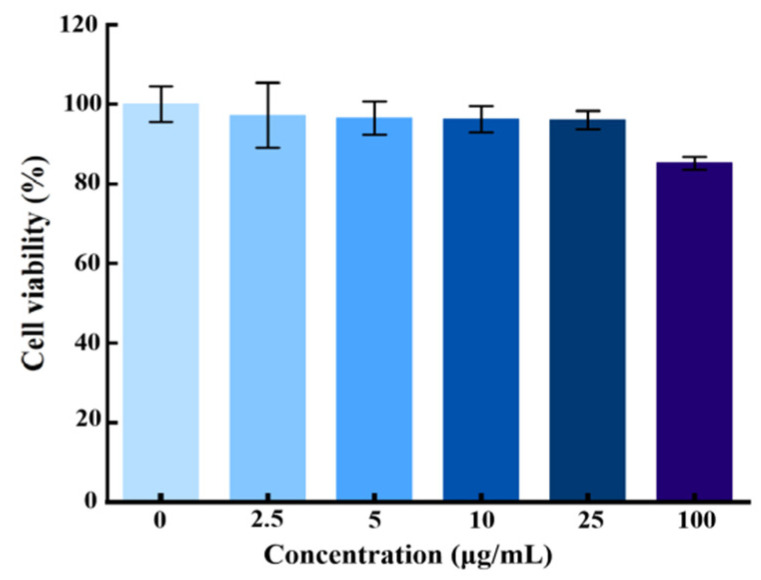
Cell viability of L929 fibroblast cells treated with different concentrations of the AgNPs@BBO-TS NE, with data expressed as mean ± SD (*n* = 3).

To evaluate the cytotoxicity of the AgNPs@BBO-TS NE, the L929 fibroblast cell was selected as a model cell, and the MTT assay was used to detect the viability activity of living cells. L929 cells were treated with different concentrations of the AgNPs@BBO-TS NE (0, 2.5, 5, 10, 25, 100 μg/mL), and the cell viability was determined after 24 h. As can be seen in Figure 7, cytotoxicity was not observed after treatment with the AgNPs@BBO-TS NE at various concentrations, and the viability of the L929 cells was still higher than 85% at the highest concentration. Above all, it was preliminarily proved that the AgNPs@BBO-TS NE was not toxic to cells.

### 2.7. Antibacterial Activity Assay

#### 2.7.1. Bacterial Inhibition Circle (BIC)

**Figure 8:**
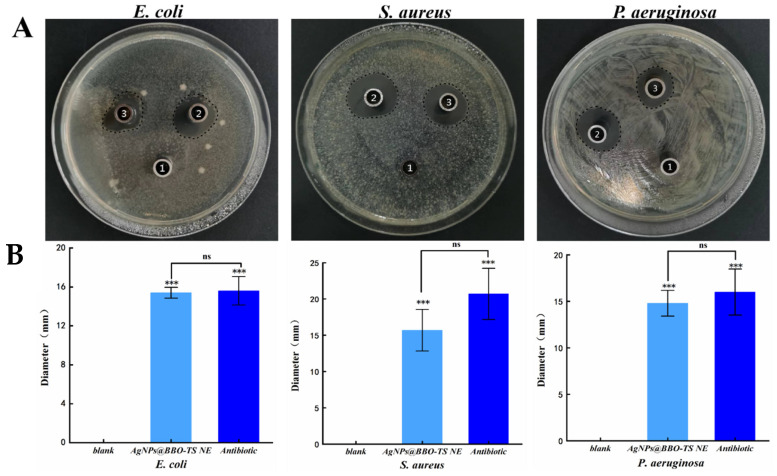
The photograph of the inhibition zones against *E. coli*, *S. aureus*, and *P. aeruginosa* (**A**) and the BIC diameter analysis (*** *p* < 0.001, as compared with saline; ns: no significant difference, as compared with antibiotics) (**B**) [1: saline (blank), 2: antibiotics, 3: AgNPs@BBO-TS NE].

The antibacterial activities of the AgNPs@BBO-TS NE were first evaluated by the Oxford cup diffusion method. The antimicrobial activity was reflected by the diameter of the bacterial inhibition circle (mm); the larger the diameter of the bacteriostatic ring, the better the bacteriostatic effect [27]. As shown in Figure 8A, the AgNPs@BBO-TS NE had a significant inhibitory effect on *S. aureus*, *E. coli* and *P. aeruginosa*. The BIC size of *E. coli* was 15.4 ± 0.55 mm, *S. aureus* was 15.7 ± 2.87 mm, and *P. aeruginosa* was 14.8 ± 1.38 mm. In *E. coli*, *S. aureus*, and *P. aeruginosa*, the inhibitory zone diameter of the AgNPs@BBO-TS NE was significantly different from that of the normal saline group (*p* values were all less than 0.001), but there was no significant difference when compared with that of an antibiotic (gentamicin sulfate, 50 μg/mL), indicating that the AgNPs@BBO-TS NE had excellent inhibitory effects on all three types of bacteria (Figure 8B). Moreover, its antibacterial performance was stable and not affected by storage time (Figure S3 and Table S3).

#### 2.7.2. Minimum Inhibitory Concentration (MIC) and Minimum Bactericidal Concentration (MBC)

The MIC is the lowest drug concentration to inhibit the visible growth of the tested bacteria, which is used to detect the sensitivity of bacteria to drugs. In the experiment, the absorbance of a nutrient broth in a 96-well plate was used to reflect the antibacterial effect of the AgNPs@BBO-TS NE. The MICs of the bacteria are shown in Table 3; the MICs of the AgNPs@BBO-TS NE to *S. aureus* and *E. coli* were 0.023 mg/mL and 0.012 mg/mL, respectively; compared with *S. aureus*, the MIC of the AgNPs@BBO-TS NE against *E. coli* was nearly onefold lower, indicating that the AgNPs@BBO-TS NE was more sensitive to inhibiting *E. coli*. The MIC of the AgNPs@BBO-TS NE against *P. aeruginosa* was 0.023 mg/mL. A broth medium was used as the negative control and a broth medium with bacterial fluid was used as the positive control. By comparing the absorbance values of the AgNPs@BBO-TS NE against different strains at MICs as well, it was found that the absorbance values at the MIC of three kinds of bacteria were quite different from those of only a broth medium plus a bacterial liquid, which had noticeable significant differences, but they were close to those of the only-broth-medium group, and there was no significant difference between this two groups, which indicated that almost all bacteria at a MIC were inhibited (Table 4).

**Figure 9:**
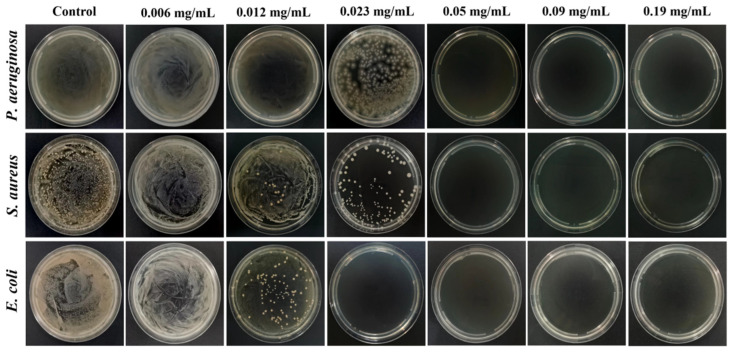
The MBCs of the AgNPs@BBO-TS NE in different concentrations on *P. aeruginosa*, *S. aureus*, and *E. coli*.

The MBC is the minimum drug concentration required to kill 99.9% of the tested microorganisms, which also reflects the bactericidal ability of drugs. Table 3 and Figure 9 displayed the MBC of the AgNPs@BBO-TS NE against three different kinds of bacteria. *S. aureus* and *P. aeruginosa* did not form colonies on the agar plate when the drug concentration was 0.05 mg/mL, and there were no colonies in the agar plate of *E. coli* after being treated with 0.023 mg/mL of the AgNPs@BBO-TS NE, which indicated that all bacteria had been killed, and the MBC of the AgNPs@BBO-TS NE against *S. aureus*, *P. aeruginosa*, and *E. coli* was determined to be 0.05, 0.05, and 0.023 mg/mL, respectively.

#### 2.7.3. Growth Curve

**Figure 10:**
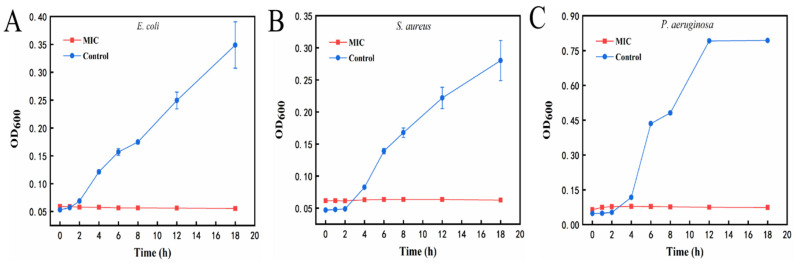
Growth curves of *E. coli* (**A**), *S. aureus* (**B**), and *P. aeruginosa* (**C**) after treatment with the MIC of the AgNPs@BBO-TS NE at different treatment times.

As shown in Figure 10A, the *E. coli* in the control group (0 mg/mL) reached the exponential growth period at 2 h and lasted until 18 h. After being treated with the MIC of the AgNPs@BBO-TS NE, the OD_600_ value of the *E. coli* did not change within 18 h. From the observation of Figure 10B,C, it could be seen that both *S. aureus* and *P. aeruginosa* in the control group reached an exponential growth period after 4 h of incubation; however, *P. aeruginosa* in the control group entered a stable period at 12 h, and the absorbance of *S. aureus* in the control group continued to increase from 4 to 18 h. However, after being treated with the MIC of the AgNPs@BBO-TS NE for 18 h, the absorbance of *S. aureus* and *P. aeruginosa* did not change, showing a trend of non-growth or negative growth. These results demonstrated that *S. aureus*, *E. coli*, and *P. aeruginosa* were very susceptible at the MIC of the AgNPs@BBO-TS NE.

#### 2.7.4. Synergy Effect

**Figure 11:**
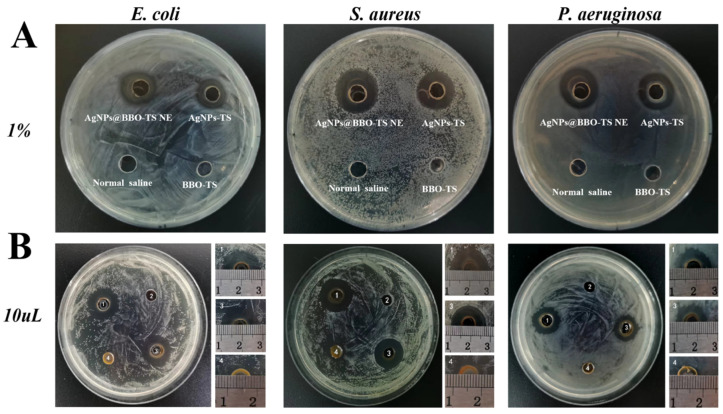
The synergistic effect of the AgNPs@BBO-TS NE against *S. aureus*, *E. coli*, and *P. aeruginosa* (**A**) (the AgNPs-TS, normal saline, and 1% BBO-TS were set as the control group); BIC graphs of 10 μL of pure BBO (**B**), 1: AgNPs@BBO-TS NE, 2: normal saline, 3: AgNPs-TS, 4: BBO.

Then, the synergy effect of different components in the AgNPs@BBO-TS NE such as the BBO, TS, and AgNPs were investigated. The diameters of the inhibition zones of the BBO-TS (containing 1% BBO), the AgNPs@BBO-TS NE, and the AgNPs-TS were analyzed, and normal saline was set as the negative control group. As shown in Figure 11A, both the AgNPs@BBO-TS NE and AgNPs-TS had growth inhibition effects on *S. aureus*, *E. coli*, and *P. aeruginosa*. The results of the bacterial inhibition circle diameter are shown in Table 5; the BIC size of the AgNPs@BBO-TS NE and AgNPs-TS was 17.0 mm and 16.5 mm on *P. aeruginosa*, 17.7 mm and 17.3 mm on *S. aureus*, and 14.1 mm and 13.3 mm on *E. coli*, respectively. The inhibition zone diameter of the AgNPs@BBO-TS NE was larger than that of the AgNPs-TS, which indicated that the inhibition ability of AgNPs@BBO-TS NE was better than AgNPs-TS solution. However, when the strains were treated with the BBO-TS, no bacteriostatic circle appeared, which may be due to the content of BBO being too low (only 0.2 μL) to kill the bacteria. To verify this hypothesis, 10 μL of pure BBO was used for a test, as shown in Figure 11B, and inhibition zones were found in *E. coli*, *S. aureus*, and *P. aeruginosa*, and the zone diameters were 10.4 mm, 11.3 mm, and 8.2 mm, respectively. The diameter of the BIC of the AgNPs@BBO-TS NE was larger than that of the pure BBO and AgNPs-TS, indicating that the different components in the AgNPs@BBO-TS NE had a good synergistic effect.

## 3. Discussion

Since the emergence of antibiotics in the early 20th century, they have been widely used due to their remarkable effects on bacteria. However, the subsequent antibiotic resistance has greatly challenged global public health and the economy [28,29]. Since ancient times, silver has been considered to have a strong antibacterial effect, and its has become the most promising metal ion to prevent bacterial infection, but silver is unstable, and the antibacterial time of the solution is short [30,31]. In recent years, many studies have found that essential oils extracted from plants, such as *Blumea balsamifera* oil, cinnamon oil, and rosemary oil, have good antibacterial activity [32,33,34]. However, their antibacterial applications are also limited by their high volatility and low water solubility.

Numerous researchers have tried to overcome these limitations by encapsulating the antimicrobial agents in colloidal delivery systems [13]. Nanoemulsion is a widely used nanoencapsulation technique that can encapsulate hydrophilic/hydrophobic active ingredients to solve their solubility, stability, and bioavailability, and it is widely used in pharmaceutical, food, and cosmetic fields [35]. The methods to produce a nanoemulsion include low-energy emulsification and high-energy emulsification. Among them, lower-energy methods require no specialized equipment, but need high concentrations of surfactants [36]. Industrially, nanoemulsions tend to be produced by high-energy emulsification methods, including high-pressure homogenization, microfluidization, and sonication [11]. High-energy emulsification methods have been used to prepare various types of plant essential oil antimicrobial nanoemulsions [22,37,38]. For example, sonication was used to create an antimicrobial nanoemulsion from *Cleome viscosa essential* oil and Tween 80, which were shown to be effective against *S. aureus*, *E. coli*, and *P. aeruginosa* [39]. In this study, we used BBO and TS as natural oil phases and emulsifiers to prepare a novel green nanoemulsion using an ultrasonic emulsification method.

To determine the optimal prescription of a BBO-TS NE, the average particle size, PDI, and zeta potential were used as indexes to investigate the BBO content, TS concentration, ultrasonic time, and ultrasonic power. After the prescription screening, we determined the optimal formulation of a BBO-TS NE was 1% BBO, 1 mg/mL of TS, an ultrasonic time of 7 min and an ultrasonic power of 385 W. Then, we used the optimal formulation to encapsulate AgNO_3_ to achieve the purpose of an efficient synergistic antibacterial. In general, the researchers will reduce the silver ions to silver nanoparticles to improve their antibacterial activity. Najafi-taher et al. [40] first prepared AgNPs by using sodium borohydride as a reducing agent and sodium citrate as a stabilizer, and then AgNPs were added to a tea tree oil nanoemulsion to obtain a TTO NE containing AgNPs. The results of the antibacterial assays showed the promising ability of the TTO NE + AgNPs for the eradication of gram-positive and gram-negative bacteria (95%). However, this method of preparing a nanoemulsion carrying AgNPs was time-consuming and complex, and it would be very exciting if AgNPs could be generated in situ while preparing nanoemulsions. Li et al. [41] showed that although TS was a non-ionic surfactant, its carboxyl and hydroxyl functional groups could form complexes with metal ions, and its hydrophilic or hydrophobic properties were conducive to the dissolution of the complexes in water. Using this property of TS, the metal elements in the soil have been successfully reduced and complexed. In the present study, in the preparation of a silver-loaded BBO-TS NE, we first added AgNO_3_ to a TS solution as an aqueous phase, and found that after AgNO_3_ was added, the solution of TS quickly (3 min) changed from yellow to brown, which attracted our attention. Through a review of the literature, it was found that the solution would turn brown when the reduction method was used to prepare AgNPs [42]. Combined with the antioxidant activity of TS, we boldly guessed that AgNPs were formed in the aqueous solution of TS. The results found through an optical microscope, DLS, SEM, EDS, and UV–visible spectroscopy confirmed the presence of AgNPs in the TS solution, the mean size of the spheroid particles was about 97.07 ± 2.88 nm, and the UV–visible spectroscopy spectra peak appeared in the region of 390–396 nm (Figure 3), which was attributed to the distinctive surface plasmon resonance peak of the AgNPs [40]. We then considered whether these AgNPs were encapsulated in the BBO-TS NE, and a peak displacement in the UV–Vis spectra of the AgNPs@BBO-TS NE was found to be between 399 nm and 411 nm (400 nm), and there was a characteristic peak of the Ag element in EDS, which proved our suspicions.

To determine the optimal amount of AgNO_3_, different concentrations of AgNO_3_ (0.5, 1, 1.5, and 2 mg/mL) were investigated. As shown in Figure 4, after adding 1.5 mg/mL of AgNO_3_, the AgNPs@BBO-TS NE had the lowest average particle size and PDI, and the droplet particle size distribution was the narrowest, indicating that 1.5 mg/mL of AgNO_3_ was the optimal concentration. Nanoemulsions have the properties of a small particle size and high dispersibility, which can make them resist gravity through Brownian motion, prevent precipitation during storage, and ensure the uniformity of the system [43]. Shamsara et al. [44] showed that when stored at room temperature for 10 d, the particle size of a nanoemulsion containing an apricot gum–pectin complex did not change significantly, which was consistent with the results of the particle size and PDI of the AgNPs@BBO-TS NE after storage for 30 d (Figure 6). On the other hand, the AgNPs@BBO-TS NE did not aggregate or precipitate after centrifugation at 4500 rpm/min for 15 min, and it had a certain centrifugal stability, which was the same as that of Li et al. [45].

After determining the best preparation technology for the AgNPs@BBO-TS NE, we evaluated its antibacterial activity. The results of the BIC, MIC, and MBC showed that the AgNPs@BBO-TS NE had excellent inhibitory effects on *E. coli*, *S. aureus*, and *P. aeruginosa* (Figure 8 and Figure 9, Table 3 and Table 4). Because the AgNPs@BBO-TS NE consisted of BBO, TS, and AgNPs, and each of these three ingredients had antibacterial activity, the synergistic antibacterial activity of the AgNPs@BBO-TS NE was also evaluated. The diameters of the inhibition zones of the BBO-TS (containing 1% BBO), AgNPs@BBO-TS NE, and AgNPs-TS were analyzed, the diameter of the BIC of the AgNPs@BBO-TS NE was larger than that of pure BBO and AgNPs-TS, indicating that the different components in the AgNPs@BBO-TS NE had a good synergistic effect (Figure 11).

Through further research and analysis, we found that compared with AgNPs, the MIC and MBC of the AgNPs@BBO-TS NE in *E. coli* was lower than that of SN-AgNPs [46], the BIC was bigger than that of the AgNPs (*E. coli*) biosynthesized by Gavade et al. [47]. And, the MIC and MBC to *S. aureus* were lower than those of three kinds of AgNPs with different particle sizes synthesized by Manosalva et al. [48]. In addition, compared with an AgNP Hydrogel, the BIC of the AgNPs@BBO-TS NE was less than that of Gel AgNPs, but the concentration of AgNO_3_ in Gel AgNPs was nearly three times higher than that of the AgNPs@BBO-TS NE [49]. Therefore, compared with other dosage forms, the AgNPs@BBO-TS NE had stronger antibacterial activity. It had been reported that the antibacterial activity of an essential oil and Ag^+^ in a nanoemulsion was enhanced because the nanoparticles had a smaller volume and a larger surface area, which could improve the release rate of Ag^+^ and, at the same time, enhance its ability to enter the bacterial cytoplasm through peptidoglycan in the cell wall and cell membrane [50].

The most crucial way for Ag^+^ and essential oils to exert their antibacterial activity is to destroy the structure of microbial cell membranes [51,52]. Therefore, we speculate that the AgNPs@BBO-TS NE will adhere to the cell membrane and enter the phospholipid bilayer structure of the bacterial cell membrane. The AgNPs in the AgNPs@BBO-TS NE combine with the negatively charged part of the membrane, creating holes in the membrane, allowing the cytoplasmic content to flow out of the cell, leading to cell death [53]. The BBO in the AgNPs@BBO-TS NE can interfere with the active transporter embedded in the phospholipid bilayer or change the integrity of the phospholipid bilayer, increase its permeability, make the cell contents leak, and lead to bacterial death [54]. In addition, the AgNPs@BBO-TS NE may cross the cell membrane and enter the cytoplasm of bacteria, which may have various effects on such bacteria [55,56]. Despite the increasing amount of research conducted on microorganisms and antibacterial mechanisms, the antibacterial mechanism of medications is multifaceted and intricate [57], the exact mechanism of the bacterial death caused by the AgNPs@BBO-TS NE is unclear, and further research is needed.

## 4. Materials and Methods

### 4.1. Strains and Media

BBO (the relative density is 0.974, L-borneol content is 43.55%) was purchased from Guizhou Aiyuan Ecology Pharmaceutical Development Co., Ltd., Guizhou, China; TS (65%) was purchased from Shanghai Yuan Ye Biology Science and Technology Co., Ltd., Shanghai, China; AgNO_3_ was obtained from Shanghai Hushi Laboratory Equipment Co., Ltd., Shanghai, China; MTT, Gentamicin sulfate, LB Broth, LB nutrient agar, and an MH(B) medium were obtained from Beijing Suolaibao Technology Co., Ltd., Beijing, China; The *S. aureus* (ATCC 25923), *E. coli* (PV1393), *P. aeruginosa* (CMCC 10104) were donated by Guizhou Medical University, Guizhou, China.

### 4.2. Preparation of BBO-TS NE

The BBO-TS NE was prepared according to the method reported by Ghosh et al. [58] with a minor modification. TS, a natural surfactant, was dissolved in ultrapure water as an aqueous phase, and added to the oil phase (BBO) drop by drop. After magnetic stirring at room temperature for 5 min, the mixture of the crude emulsion was treated with a JY92-IIN ultrasonic homogenizer (Ningbo Scientz Biotechnology Co., Ltd., Ningbo, China) using the following homogenizing conditions: an ultrasound time of 2 min with an interval of 5 s on and 5 s off for an ice bath with an ultrasound power of 162 W.

### 4.3. Single-Factor Experiments to Optimize the Prescription of the BBO-TS NE

The single-factor method was used to investigate the influence of various factors on the BBO-TS NE [59], including the mass fraction of BBO (0.5%, 1%, 3%, 5%), the concentration of TS (0.75, 1, 1.25, 1.5, 1.75 mg/mL), the ultrasonic time (1, 2, 3, 5, 7, 10 min), and the ultrasonic power (65, 162, 260, 358, 455 W). With the average particle size as the main evaluation index and the polydispersity index (PDI) and zeta potential as the secondary evaluation indexes, the formulation and technological conditions of the nanoemulsion were screened and optimized.

### 4.4. The Existing Form of Silver in the BBO-TS NE

After preparing the TS solution (1 mg/mL), AgNO_3_ at a concentration of 1 mg/mL was added to obtain a Ag-TS mixture solution, and the appearance changes before and after adding AgNO_3_ were recorded. A scanning electron microscope (SEM, S-4800, Hitachi, Japan), an EDS spectrum, an optical microscope (XSP-C204, Chongqing Chongguang Industry Co., Ltd., Chongqing, China), and dynamic light scattering (DLS) were used to evaluate the formation of particles in the TS solution, and the spectral response of this mixture’s solution at 300–800 nm was recorded by a UV–visible spectrophotometer.

The obtained AgNPs-TS mixture solution was added to 1% BBO drop by drop, after continuously stirring for 5 min with a magnetic mixer, then undergoing ultrasonic emulsification for 7 min using the optimal ultrasound conditions of preparation for a BBO-TS NE, scanning its UV–visible spectrum within 300–800 nm, and finally, the EDS was determined.

### 4.5. Determination of the AgNO_3_ Concentration

Silver was loaded in an optimal prescription to the BBO-TS NE. AgNO_3_ was added in 1.0 mg/mL of a TS aqueous solution to be prepared into aqueous phases with different concentrations (0.5, 1.0, 1.5, 2 mg/mL of AgNO_3_). Then, the aqueous phases and 1% BBO were intensively mixed for 5 min with a magnetic mixer. This crude emulsion was treated for 7 min with intermittent intervals for 5 s of ultrasonic crushing at 358 W. The average particle size, zeta potential, and PDI were measured to determine the optimal concentration of AgNO_3_.

### 4.6. Characterization of the AgNPs@BBO-TS NE

#### 4.6.1. Appearance and Type Analysis

A batch of AgNPs@BBO-TS NEs was prepared, and their appearance was photographed and observed. The type of AgNPs@BBO-TS NE was identified using the dyeing method. AgNPs@BBO-TS NEs were prepared according to the optimal formula, and the same amount of Sudan red (an oil-soluble dye) and methylene blue (a water-soluble dye) were added dropwise, respectively, and the diffusion speeds of these two dyes in the AgNPs@BBO-TS NEs were compared.

#### 4.6.2. Average Particle Size, Zeta Potential, Particle Size Distribution, and PDI

The AgNPs@BBO-TS NEs were diluted at the ratio of 1:40 using deionized water, a certain amount of diluted sample was put into the sample pool, and the particle size, zeta potential, and PDI of the AgNPs@BBO-TS NEs were determined via the dynamic light scattering instrument’s measurement chamber (Beckman nanoscale particle size analyzer DelsaMax PRO, Beckman Coulter, Inc., Bria, CA, USA) [60].

#### 4.6.3. Morphological Analysis

The AgNPs@BBO-TS NE was diluted 40 times with deionized water, and a drop of the sample was added to a slide glass surface, the cover glass was gently covered at an inclination of 45°, put under an optical microscope with the background light adjusted appropriately, the morphology and dispersity of the AgNPs@BBO-TS NE droplets were observed, and photos were taken [61]. Furthermore, 200 µL of the diluted AgNPs@BBO-TS NE was dripped onto the surface of the copper mesh. After 1.5–2 min, the excess liquid was absorbed by filter paper, and the phosphotungstic acid solution was dripped for negative dyeing. After natural drying, the copper mesh was placed under the transmission electron microscope (TEM, Hitachi, Tokyo, Japan) to observe the morphology of the AgNPs@BBO-TS NE.

#### 4.6.4. Determination of pH Value

At room temperature, the pH of the AgNPs@BBO-TS NE was determined using a pH meter (PHS-3E, Shanghai Yi Electrical Scientific Instruments Co., Ltd., Shanghai, China). The experiment was repeated three times, and the result is expressed as the mean ± standard deviation.

#### 4.6.5. Determination of Turbidity

The prepared AgNPs@BBO-TS NE was diluted 40 times with PBS, the absorbance was measured by a UV–visible spectrophotometer at 600 nm, and its turbidity was calculated according to the following formula [62]:

$$T=2.302\times \frac{A\times V}{I}$$

where *A* is the absorbance of the diluted AgNPs@BBO-TS NE at a 600 nm wavelength, *V* is the dilution multiple, and *I* is the optical path difference of 1 cm.

### 4.7. Stability

The stability study of the AgNPs@BBO-TS NE in terms of its phase separation, changes in its average particle size, and PDI was evaluated via centrifugation and storage at room temperature for 30 d. The AgNPs@BBO-TS NE was diluted 10 times with 95% ethanol, and the absorbance was measured at 425 nm using an ultraviolet spectrophotometer (*A*_0_). At the same time, 2 mL of the sample was sucked into a centrifuge tube, centrifuged at 4500 rpm/min for 15 min, the supernatant was diluted with 95% ethanol 10 times, and the absorbance at 425 nm was measured (*A*_1_). The appearance of the nanoemulsion before and after centrifugation was observed, and the centrifugal stability coefficient (*K*) was calculated according to the change of absorbance before and after centrifugation. The centrifugal stability coefficient (*K*) was calculated as follows [63]:

*K* = (*A*_1_/*A*_0_) × 100%

*A*_0_ and *A*_1_ were the absorbance values at 425 nm of the AgNPs@BBO-TS NE before and after centrifugation, respectively.

To study its long-term stability, AgNPs@BBO-TS NE samples were stored at room temperature for 30 d, sampled every 10 d to detect the average particle size and PDI, and each measurement was repeated three times.

### 4.8. Cytotoxicity

The cytotoxicity of the AgNPs@BBO-TS NE was studied using an MTT assay. The L929 fibroblast cells were seeded on a 96-well plate at a density of 5 × 10^4^ cells/well. After 24 h, the cells were exposed to a series of AgNPs@BBO-TS NEs in different concentrations (0, 2.5, 5, 10, 25, and 100 μg/mL), and the complete culture medium was set as a blank control group. After incubation for another 24 h, 20 μL of MTT solution (5 mg/mL) was added to each well. After 4 h, the culture solution was sucked out, and 100 μL of DMSO was added and mixed thoroughly, the absorbance was measured at 570 nm using a microplate spectrophotometer (Thermo Scientific™ Multiskan Sky, Thermo Fisher Scientific Shier Science & Technology Company, Waltham, MA, USA). The cell viability was calculated by the following equation [64]:

$$Cell\;viability(\%)=\frac{ODsample}{ODcontrol}\times 100\%$$

### 4.9. Antimicrobial Activity

#### 4.9.1. Measurement of Bacterial Inhibition Circles

The inhibition effect of the AgNPs@BBO-TS NE on *E. coli*, *S. aureus*, and *P. aeruginosa* was determined via the Oxford cup diffusion method. An amount of 500 μL of bacterial suspension (1 × 10^6^ CFU/mL) was evenly coated on the LB agar plate with an L-shaped coating rod. The AgNPs@BBO-TS NEs were used as the experimental group, normal saline as the blank control group, and gentamicin sulfate (50 μg/mL) as the positive control group. Oxford cups (with an inner diameter of 6 mm) were placed on the culture medium, and a 100 μL liquid test sample was added to each Oxford cup. Three groups of parallel experiments were conducted for each strain, and the diameter of the bacteriostatic circle was observed and measured after culturing for 18 h at 37 °C [65].

#### 4.9.2. Minimum Inhibitory Concentration (MIC) and Minimum Bactericidal Concentration (MBC)

The micro-broth dilution method measured the minimum inhibitory concentration (MIC) of the AgNPs@BBO-TS NE. An amount of 100 μL of sterilized nutrient broth culture medium was added to each well of the 96-well plate, and 100 μL of the AgNPs@BBO-TS NE was added to the first well; the mixture solution was diluted sequentially to the tenth well using a twofold dilution method, discarding 100 μL, and then adding 100 μL of bacterial suspension with a concentration of 1 × 10^6^ CFU/mL to each well. Each row of 10 wells was set as the experimental group, each strain was repeated three times, cultured at 37 °C for 24 h; the absorbance was determined at 600 nm. The negative control group in the 11th row of the 96-well plate was added to the broth medium. At the same time, the positive control group in the 12th row of the 96-well plate was added to the culture medium and the bacterial liquid [66].

According to the experimental method reported by Luo et al. with a slight modification [67], AgNPs@BBO-TS NEs with concentrations of 0.006, 0.012, 0.023, 0.05, 0.09, and 0.19 mg/mL were used as the experimental group, while the control group only contained bacteria and a culture medium. An amount of 100 μL of mixed solution of AgNPs@BBO-TS NEs with different concentrations were dripped onto an agar plate, coated evenly, and incubated for 20 h at 37 °C. The lowest sample concentration without colony growth (killing ≥ 99.9% of colonies) was defined as the MBC value.

#### 4.9.3. Growth Curve

To further determine the antibacterial activity of the AgNPs@BBO-TS NE, a mixture of the AgNPs@BBO-TS NE and the medium was diluted to the MIC of three bacteria, and then 100 μL of bacterial suspension was added. OD values at 600 nm were measured at intervals of 0, 1, 2, 4, 6, 8, 12, and 18 h, respectively [68].

#### 4.9.4. Synergy Effect

The synergistic effects of the AgNPs@BBO-TS NE on different strains were determined using the punching method [69], with the normal saline and the same concentration of the AgNPs-TS and BBO (dissolved in TS solution, 1%) were set as control groups. An inoculated 200 μL of bacterial suspension (1 × 10^6^ CFU/mL) was placed on the culture plate and coated evenly using an L-shaped coating rod. Then, four holes (with an inner diameter of 6 mm) were made on the agar plate, and 20 μL of different liquid test samples were placed into each hole. After culturing for 18 h at 37 °C, the inhibition zones were measured and recorded. At the same time, the inhibition zone of 10 μL of pure BBO was also detected.

### 4.10. Statistical Analysis

All experiments were determined at least three times with freshly prepared samples, and the results were reported as the average and standard deviation of these measured values. SPSS 26.0 software was used for the one-way analysis of variance (ANOVA) to determine the significant differences. When *p* < 0.05, the difference is considered statistically significant, * *p* < 0.05, ** *p* < 0.01, *** *p* < 0.001.

## 5. Conclusions

In this present study, a novel green AgNPs@BBO-TS NE with an excellent antibacterial effect was successfully prepared using BBO as an oil phase and TS as a natural emulsifier and reducing agent, and their physicochemical properties and in vitro antimicrobial activity were also evaluated systematically. The optimal formula of the AgNPs@BBO-TS NE was as follows: 1% BBO, 1 mg/mL of TS, 358 W of ultrasonic power, 7 min of ultrasonic time, and 1.5 mg/mL of AgNO_3_. The average particle size of the AgNPs and the AgNPs@BBO-TS NE were 97.07 ± 2.88 nm and 249.47 ± 6.23 nm, respectively. The AgNPs@BBO-TS NE exhibited good stability after storing for 30 d at room temperature. The combination of BBO, TS, and AgNPs in the AgNPs@BBO-TS NE had shown noticeable antibacterial effects in vitro on *E. coli*, *S. aureus*, and *P. aeruginosa*. We believe that the synthesized AgNPs@BBO-TS NE has the potential to be an eco-friendly antimicrobial agent in the biomedical field. Moreover, this experiment confirmed the feasibility of using TS as a natural surfactant and reducing agent to rapidly synthesize new AgNP-loaded green nanoemulsions in one step, and provided a basis and reference for expanding the application of TS in materials science, life science, and medicine fields. However, the antibacterial mechanisms of the AgNPs@BBO-TS NE need to be studied further.

## Figures and Tables

**Table 1:** The particle size, PDI, and zeta potential of the AgNPs@BBO-TS NE.

Sample	Particle Size (nm)	PDI	Zeta Potential (mV)
AgNPs@BBO-TS NE	249.47 ± 6.23	0.239 ± 0.003	−35.82 ± 4.26

**Table 2:** The results of the centrifugation stability, with data given as mean ± SD, *n* = 3.

Batch Number	*A*_0_	*A*_1_	*K*
1	0.511 ± 0.040	0.469 ± 0.001	91.8%
2	0.458 ± 0.002	0.437 ± 0.001	95.6%
3	0.502 ± 0.002	0.471 ± 0.001	93.8%

**Table 3:** The MICs and MBCs of the AgNPs@BBO-TS NE, with data given as mean ± SD, *n* = 3.

Culture	MIC (mg/mL)	MBC (mg/mL)
*E. coli*	0.012	0.023
*S. aureus*	0.023	0.05
*P. aeruginosa*	0.023	0.05

**Table 4:** A comparison of the OD600 of three groups under different strains.

Group Number	*E. coli*	*S. aureus*	*P. aeruginosa*
MIC	0.07 ± 0.010 ^b^	0.07 ± 0.003 ^b^	0.07 ± 0.004 ^b^
Negative	0.05 ± 0.002 ^b^	0.06 ± 0.003 ^b^	0.07 ± 0.006 ^b^
Positive	0.46 ± 0.021 ^a^	0.37 ± 0.019 ^a^	1.04 ± 0.011 ^a^

^a^ and ^b^ indicate the significant difference among the three experimental groups for each strain (*p* < 0.001).

**Table 5:** The synergistic effect evaluation of the AgNPs@BBO-TS NE, with BIC diameter data given as mean ± SD, *n* = 3.

Group Number	Strain	Bacteriostatic Ring Diameter (mm)			
		AgNPs@BBO-TS NE	AgNPs-TS	BBO-TS/BBO	Normal Saline
BBO-TS (1%)	*E. coli*	14.1 ± 0.63	13.3 ± 0.47	——	——
	*S. aureus*	17.7 ± 1.00	17.3 ± 0.24	——	——
	*P. aeruginosa*	17.0 ± 0.82	16.5 ± 0.39	——	——
BBO (10 μL)	*E. coli*	19.4 ± 0.81	18.8 ± 0.49	10.4 ± 0.13	——
	*S. aureus*	20.6 ± 0.71	18.2 ± 0.47	11.3 ± 0.48	——
	*P. aeruginosa*	17.3 ± 0.92	16.0 ± 0.70	8.2 ± 0.30	——

## Data Availability

The original contributions presented in the study are included in the article, and further inquiries can be directed to the corresponding author.

## Supplementary Materials

The following supporting information can be downloaded at: https://www.mdpi.com/article/10.3390/molecules29092009/s1, Figure S1: EDS elemental composition analysis of AgNPs-TS solution; Figure S2: EDS elemental composition analysis of BBO-TS NE-2; Figure S3: Pictures of inhibitory zones of AgNPs@BBO-TS NE on *E. coli*, *S. aureus*, and *P. aeruginosa* in different storage times (bacterial suspension 200 μL, AgNPs@BBO-TS NE 20 μL); Table S1: Elemental composition analysis of AgNPs-TS solution; Table S2: Elemental composition analysis of BBO-TS NE-2; Table S3: BIC diameter of AgNPs@BBO-TS NE with different storage time to *E. coli*, *S. aureus* and *P. aeruginosa* (bacterial suspension 200 μL, AgNPs@BBO-TS NE 20 μL).
