# Supplementary material for: Green Preparation and Antibacterial Activity Evaluation of AgNPs-Blumea balsamifera Oil Nanoemulsion
Source: Molecules. 2024 Apr 26;29(9):2009. doi: 10.3390/molecules29092009 (PMC11085303; doi:10.3390/molecules29092009)
Supplement: Supplementary file 1 [file molecules-29-02009-s001.zip › molecules-2908142-supplementary.pdf]

Supplementary Materials

Green preparation and antibacterial activity evaluation of AgNPs-*Blumea balsamifera* oil nanoemulsion

Chunfang Ma, Bingnan Liu, Lingfeng Du, Wei Liu, Yue Zhu, Teng Chen, Zuhua Wang, Hongpeng Chen and Yuxin Pang

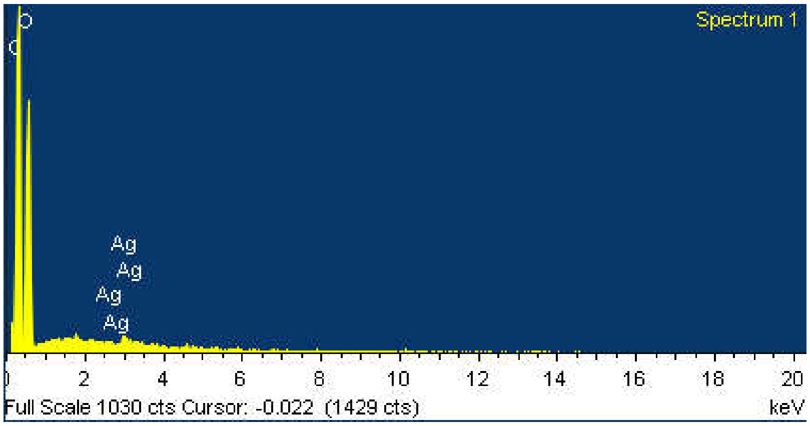

Figure S1. EDS elemental composition analysis of AgNPs-TS solution.

Table S1. Elemental composition analysis of AgNPs-TS solution.

Element	Weight%	Atomic%
C K	44.51	52.01
O K	54.56	47.86
Ag L	0.93	0.12
Totals	100.00	

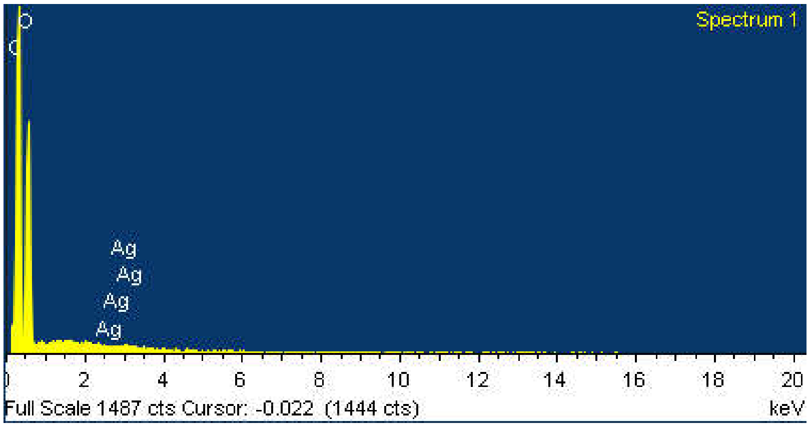

Figure S2. EDS elemental composition analysis of BBO-TS NE-2.

Table S2. Elemental composition analysis of BBO-TS NE-2.

Element	Weight%	Atomic%
C K	46.35	53.72
O K	53.13	46.22
Ag L	0.52	0.07
Totals	100.00	

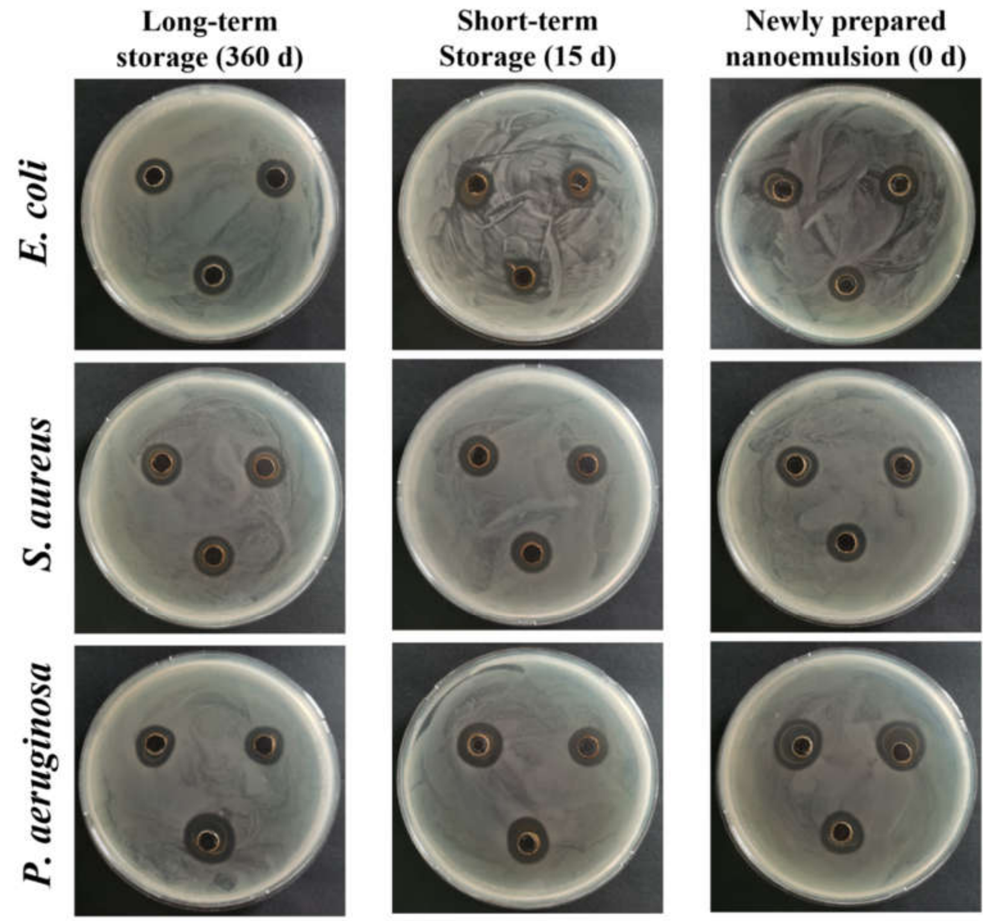

Figure S3. Pictures of inhibitory zones of AgNPs@BBO-TS NE on *E. coli*, *S. aureus*, and *P. aeruginosa* in different storage times (bacterial suspension 200 μL, AgNPs@BBO-TS NE 20 μL).

Table S3. BIC diameter of AgNPs@BBO-TS NE with different storage time to *E. coli*, *S. aureus*, and *P. aeruginosa* (bacterial suspension 200 μL, AgNPs@BBO-TS NE 20 μL).

Culture/Group	Bacteriostatic ring diameter (mm)		
	Long-term storage (360 d)	Short-term Storage (15 d)	Newly prepared nanoemulsion (0 d)
E. coli	13.3 ± 0.51	13.2 ± 0.54	13.5 ± 0.41
S. aureus	14.3 ± 0.24	14.8 ± 0.38	14.6 ± 0.85
P. aeruginosa	15.6 ± 1.73	15.4 ± 0.96	16.9 ± 1.59
